# Supplementary material for: Small-Molecule Anti-HIV-1 Agents Based on HIV-1 Capsid Proteins
Source: Biomolecules. 2021 Feb 3;11(2):208. doi: 10.3390/biom11020208 (PMC7913237; doi:10.3390/biom11020208)
Supplement: Supplementary file 1 [file biomolecules-11-00208-s001.pdf]

__Supplementary Materials__

Small-molecule anti-HIV-1 agents based on HIV-1 capsid proteins

Takuya Kobayakawa[a], Masaru Yokoyama[b], Kohei Tsuji[a], Masayuki Fujino[c], Masaki Kurakami[a], Sayaka Boku[a], Miyuki Nakayama[a], Moemi Kaneko[a], Nami Ohashi[a,$], Osamu Kotani[b], Tsutomu Murakami[c,*], Hironori Sato[b,*], Hirokazu Tamamura[a,*]

[a]*Institute of Biomaterials and Bioengineering, Tokyo Medical and Dental University, Chiyoda-ku, Tokyo 101-0062, Japan*

[b]*Pathogen Genomics Center, National Institute of Infectious Diseases, Musashimurayama, Tokyo 208-0011, Japan*

[c]*AIDS Research Center, National Institute of Infectious Diseases, Shinjuku-ku, Tokyo 162-8640, Japan*

[$]*present address: Showa Pharmaceutical University, Machida, Tokyo 194-8543, Japan*

Contents:

Experimental procedures including synthesis and characterization data of compounds, *in silico* screening of antiviral candidates and evaluation of anti-HIV activity and cytotoxicity

Experimental Procedures

Synthesis of MKN-1 (1) and its diasteomer

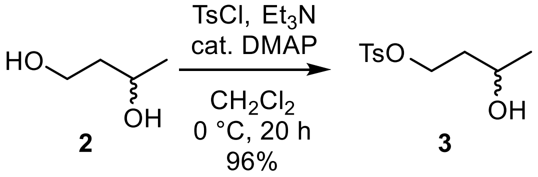

3-Hydroxybutyl 4-methylbenzenesulfonate (3)[67]

To a solution of 1,3-butandiol (**2**) (901 mg, 10.0 mmol) in CH_2Cl_2 (20 mL) were added DMAP (24.4 mg, 0.200 mmol), Et_3N (4.18 mL, 300 mmol) and TsCl (2.29 g, 12.0 mmol) at 0 °C. After the reaction mixture was stirred for 20 h at this temperature, the mixture was quenched by H_2O and extracted with CH_2Cl_2, then washed with brine. The combined organic layer was dried over $MgSO_4$, then concentrated under reduced pressure, followed by purification with column chromatography (hexane/EtOAc = 1:1) to provide the title compound **3** (2.36 g, 96% yield) as colorless oil. ^{1}H-NMR and ESI-MS data are consistent with those of the previous paper.[67]

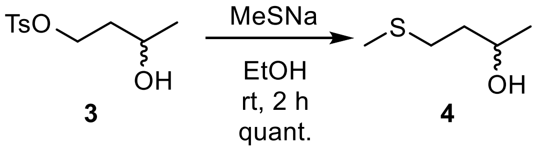

4-(Methylthio) butan-2-ol (4)

To a solution of **3** (489 mg, 2.00 mmol) in EtOH (12 mL) was added MeSNa (210 mg, 3.00 mmol). After the reaction mixture was stirred at room temperature for 2 h, the mixture was quenched by H_2O and extracted with CH_2Cl_2 and EtOAc, then washed with brine. The combined organic layer was dried over $MgSO_4$, then concentrated under reduced pressure, followed by purification with column chromatography (hexane/EtOAc = 1:1) to provide the title compound **4** (240 mg, quantitative) as yellow oil.

^{1}H-NMR (500 MHz, $CDCl_3$) 1.197–1.21 (m, 3H), 1.70–1.74 (m, 2H), 2.10 (s, 3H), 2.585–2.614 (m, 2H), 3.899–3.96 (m, 1H); ^{13}C NMR (125 MHz, $CDCl_3$) 15.5, 23.5, 30.9, 37.8, 67.2; HRMS (ESI), *m*/*z* calcd for $C_5H_{12}OS$ $[M + H]^+$ 121.0682, found 121.0682.

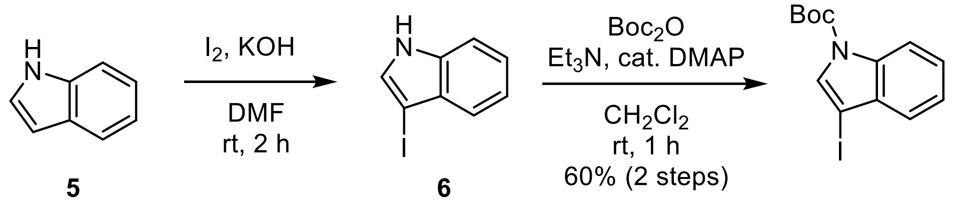

5 6 7

***tert*-Butyl 3-iodo-1H-indole-1-carboxylate (7)**[68]

Indole (**5**) (2.34 g, 20.0 mmol) and KOH (2.81 g, 50.0 mmol) were combined in DMF (50.0 mL) and stirred at room temperature for 20 min. The mixture was cooled to 0 °C, and I_2 (5.08 g, 20.0 mmol) was added, then stirred for 15 min at 0 °C. After the reaction mixture was stirred at room temperature for 2 h, the mixture was quenched by saturated aqueous $Na_2S_2O_3$ and extracted with EtOAc. The combined organic layer was washed with saturated aqueous $Na_2S_2O_3$, dried over $MgSO_4$, then concentrated under reduced pressure and used without purification for the next step. The crude product (**6**) was dissolved in CH_2Cl_2 (100 mL), and Et_3N (8.36 mL, 60.0 mmol), DMAP (244 mg, 2.00 mmol) and Boc_2O (5.20 g, 24.0 mmol) were added to the solution. After the reaction stirred at room temperature for 2 h, the mixture was quenched by saturated aqueous $Na_2S_2O_3$ and extracted with CH_2Cl_2, then washed with brine. The combined organic layer was dried over $MgSO_4$, then concentrated under reduced pressure, followed by purification with column chromatography (hexane/EtOAc = 20:1) to provide the title compound **7** (4.12 g, 60% yield) as orange oil. ^{1}H-NMR and ESI-MS data are consistent with those of the previous paper.[68]

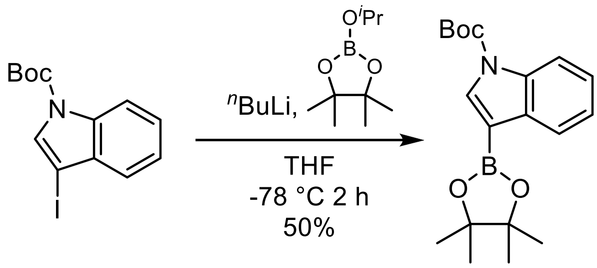

7 8

***tert*-Butyl 3-(4,4,5,5-tetramethyl-1,3,2-dioxaborolan-2-yl)-1H-indol-2-carboxylate (8)**

To a solution of **7** (2.61 g, 7.00 mmol) in THF (40 mL) was added *n*BuLi (11.9 mL of a 0.999 M solution, 11.9 mmol) dropwise over 10 min at -78 °C. The reaction mixture was stirred at -78 °C for 30 min, then isopropoxypinacol boronate (2.80 mL, 14.0 mmol) was added dropwise over 5 min. After the reaction mixture was stirred for 2 h at this temperature, the mixture was quenched by saturated aqueous NH_4Cl and extracted with Et_2O, then washed with H_2O and brine. The combined organic layer was dried over $MgSO_4$, then concentrated under reduced pressure, followed by purification with column chromatography (hexane/EtOAc = 20:1) to provide the title

compound **8** (1.19 g, 50%) as off-white solid. ^{1}H-NMR and ESI-MS data are consistent with those of the previous paper.[68]

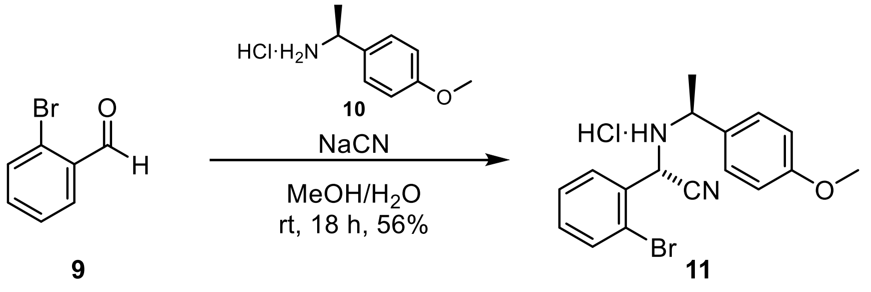

(*S*,*S*)-2-(2-Bromophenyl)-2-[1-(4-methoxyphenyl)ethylamino]acetonitrile hydrochloride (11)[69]

(*S*)-1-(4-Methoxyphenyl) ethylamine hydrochloride (**10**) (1.25 g, 6.66 mmol) and NaCN (342 mg, 6.99 mmol) were dissolved in H_2O (9 mL). To the mixture was then added 2-bromobenzaldehyde (**9**) (0.770 mL, 6.66 mmol) in MeOH (9 mL), and the reaction mixture was stirred for 18 h. The mixture was then diluted with H_2O, and the resulting solid was collected via filtration and washed with hexane. The crude solid was dissolved in Et_2O and re-precipitated from Et_2O/hexane to provide the title compound **11** (1.43 g, 56%) as white plates.

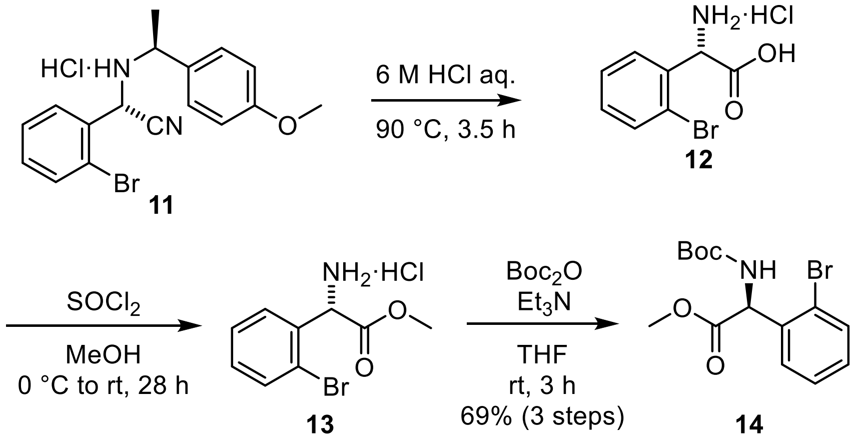

Methyl (*S*)-2-(2-bromophenyl)-2-((*tert*-butoxycarbonyl) amino) acetate (14)

Compound **11** (1.20 g, 3.11 mmol) was treated with 6 M aqueous HCl (36.7 mL), and the mixture was heated at 90 °C for 3.5 h. The reaction mixture was allowed to cool to room temperature and extracted with Et_2O. The organic layer was discarded, and the aqueous layer was concentrated under reduced pressure. The residue was freeze-dried to provide the crude mixture of **12** as white solid. The mixture was dissolved in MeOH (15 mL), and $SOCl_2$ (1.35 mL, 18.6 mmol) was added dropwise at 0 °C. After the reaction mixture stirred at room temperature for 28 h, evaporated to provide the crude mixture of **13** as yellow solid. The mixture was then dissolved in THF (15 mL), and Et_3N (0.860 mL, 6.20 mmol) was added at 0 °C. The mixture was allowed to warm to room

temperature and stirred 15 min, then Boc_2O (812 mg, 3.72 mmol) was added. After the reaction mixture stirred at room temperature for 3 h, evaporated and the residue was then diluted with H_2O and extracted with CH_2Cl_2, then washed with saturated aqueous NH_4Cl and brine. The organic layer was dried over $MgSO_4$, then concentrated under reduced pressure, followed by purification with column chromatography (hexane/EtOAc = 4:1) to provide the title compound **14** (732 mg, 69%, 3 steps) as yellow oil.

[$]_D$ +80.3 (*c* 1.01, $CHCl_3$); IR(ATR) ν 1711 (CO), 1746 (CO) cm^{-1}; ^{1}H-NMR (500 MHz, $CDCl_3$): 1.43 (s, 9H), 3.73 (s, 3H), 5.64–5.72 (m, 1H), 7.17–7.59 (m, 4H); ^{13}C-NMR (125 MHz, $CDCl_3$): 28.4, 53.1, 57.7, 80.4, 123.8, 128.0, 129.9, 130.0, 133.6, 137.0, 154.9, 171.3; HRMS (ESI), *m*/*z* calcd for $C_{18}H_{29}N_2O_2S$ $[M+H]^+$ 366.0311, found 366.0311.

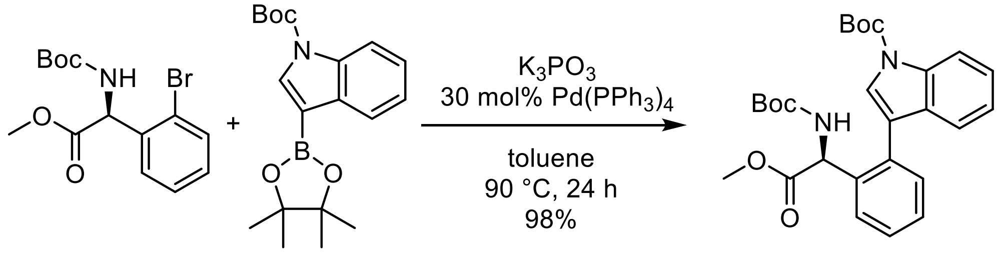

14 **8** **15**

tert-Butyl (_S_)-3-(2-(1-((_tert_-butoxycarbonyl)amino)-2-methoxy-2-oxoethyl)phenyl)-1_H_-indole-1-carboxylate (15)

Compound **14** (34.4 mg, 0.100 mmol), compound **8** (85.8 mg, 0.250 mmol), K_3PO_4 (127 mg, 0.600 mmol) and $Pd(PPh_3)_4$ (34.6 mg, 30.0 mol) were combined in toluen (3 mL), and the reaction mixture was then stirred at 90 °C for 24 h. After being cooled to room temperature, the mixture was filtered through celite to remove palladium. The filtrate was evaporated, and the residue was purified by column chromatography (hexane/EtOAc = 5:1) to provide the title compound **15** (46.9 mg, 98% yield) as yellow solid.

[$]_D$ +54.0 (*c* = 1.01, $CHCl_3$); IR(ATR) ν 1732 (CO), 1714 (CO) cm^{-1}; ^{1}H-NMR (500 MHz, $CDCl_3$) 1.37 (s, 9H), 1.69 (s, 9H), 3.59 (s, 3H), 5.29–5.55 (m, 1H), 7.21–7.48 (m, 7H), 7.82 (s, 1H), 8.22–8.23 (m, 1H); ^{13}C-NMR (125 MHz, $CDCl_3$) 28.4 (6C), 52.7, 54.9, 80.1, 83.9, 115.4, 119.3, 120.0, 122.9, 124.8, 125.1, 127.5, 128.4, 128.5, 130.8, 131.8, 133.4, 135.5, 136.1, 149.8, 154.8, 172.2; HRMS (ESI), *m*/*z* calcd for $C_{27}H_{33}N_2O_6$ $[M+H]^+$ 481.2333, found 481.2329.

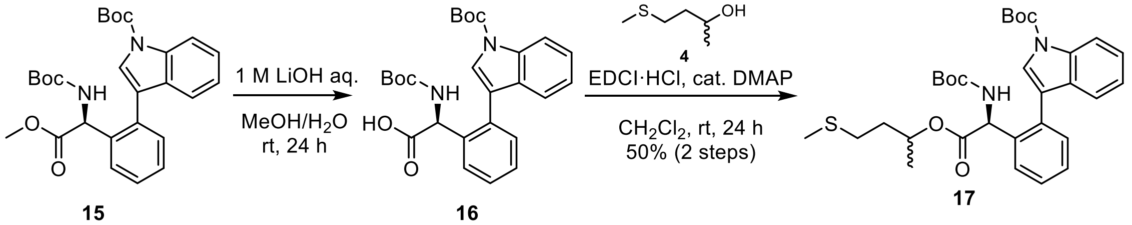

***tert*-Butyl 3-(2-((8*S*)-5,12,12-trimethyl-7,10-dioxo-6,11-dioxa-2-thia-9-azatridecan-8-yl)phenyl)-1*H*-indole-1-carboxylate (17)**

To a solution of **15** (48.0 mg, 0.100 mmol) in MeOH (0.1 mL) was added 1 M aqueous LiOH (0.400 mL) dropwise at 0 °C. After the reaction mixture was stirred at room temperature, the mixture was quenched by saturated aqueous NH_4Cl (pH = 6) and extracted with EtOAc, then washed with brine. The combined organic layer was dried over $MgSO_4$, then concentrated under reduced pressure to provide the crude mixture of **16**. The crude mixture was dissolved in CH_2Cl_2 (1 mL), and EDCI·HCl (28.8 mg, 0.150 mmol), DMAP (1.22 mg, 10.0 mol) and **4** (18.0 mg, 0.150 mmol) in CH_2Cl_2 (0.5 mL) were added at 0 °C. After the reaction mixture was stirred at room temperature for 24 h, the mixture was quenched by saturated aqueous $NaHCO_3$ and extracted with CH_2Cl_2, then washed with brine. The combined organic layer was dried over $MgSO_4$, then concentrated under reduced pressure to provide the title crude compound **17** (28.4 mg) as yellow oil, which was used for the next step without further purification.

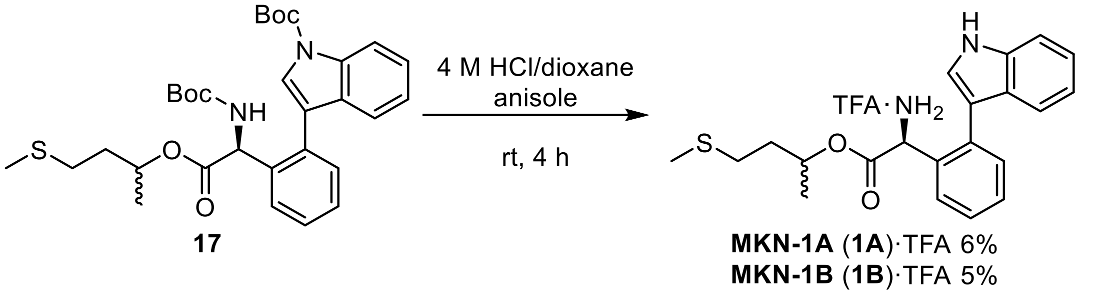

4-(Methylthio)butan-2-yl (2*S*)-2-(2-(1*H*-indol-3-yl)phenyl)-2-aminoacetate MKN-1A (1A)/MKN-1B (1B)

To a mixture of a crude compound **17** (28.4 mg, 50.0 mol) and anisole (22.0 μL, 200 mol) was slowly added 4 M HCl/dioxane (0.250 mL, 1.00 mmol) at 0 °C. The reaction mixture was stirred at room temperature for 4 h, then concentrated under reduced pressure, followed by purification with HPLC to provide the trifluoroacetate (TFA) salt of the title compounds MKN-1A (**1A**)/MKN-1B (**1B**) (1.45 mg, 3.00 mol, 3%/1.21 mg, 2.51 mol, 3% in 3 steps) as yellow solid. (**1A** and **1B**, the order of HPLC retention time)

^{1}H-NMR (500 MHz, MeOH-d_4) 1.26–1.27 (m, 3H), 1.29–1.34 (m, 3H), 1.73–1.79 (m, 2H), 2.00–2.06 (m, 1H), 2.12–2.17 (m, 1H), 5.09–5.12 (m, 1H), 5.38 (s, 1H), 7.07–7.11 (m, 1H), 7.19–7.22 (m, 1H), 7.43 (s, 1H), 7.47–7.51 (m, 3H), 7.45–7.61 (m, 3H); ^{13}C NMR (125 MHz, $CDCl_3$) 15.2, 20.0,

30.3, 36.0, 54.3, 74.1, 112.7, 114.6, 119.8, 121.0, 123.3, 125.5, 127.4, 128.4, 131.0, 132.7, 133.7, 137.9, 138.0, 167.0; HRMS (ESI), *m*/*z* calcd for $C_{21}H_{24}N_2O_2S$ $[M+H]^+$ 369.1631, found 369.1636.

Stereoselective synthesis of MKN-1 (1)

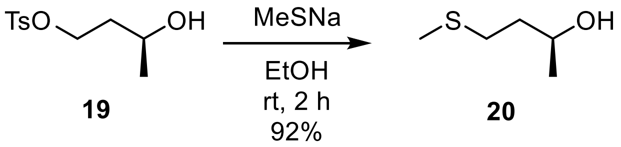

(*S*)-4-(Methylthio) butan-2-ol (20)

Alcohol **19** was synthesized using the previously reported procedure.[70] To alcohol **19** (1.44 g, 5.45 mmol) in EtOH (27 mL) was added MeSNa (458 mg, 6.54 mmol) at room temperature under argon. After the reaction mixture was stirred at room temperature for 2 h, the mixture was quenched by H_2O and extracted with EtOAc. The organic layer was dried over $MgSO_4$, then concentrated under reduced pressure, followed by purified by flash column chromatography (hexane/EtOAc = 4:1) to provide the title compound **20** (60.4 mg, 92% yield) as colorless oil: [ ]$_D$ +29.7 (*c* 1.02, $CHCl_3$); IR(ATR) ν 3364 (OH) cm^{-1}; ^{1}H-NMR (500 MHz, $CDCl_3$) 1.22 (d, *J* = 6.5 Hz, 3H), 1.72–1.76 (m, 2H), 2.12 (s. 3H), 2.62 (t, *J* = 7.3 Hz, 2H), 3.92–3.98 (m, 1H); ^{13}C-NMR (125 MHz, $CDCl_3$) 15.6, 23.7, 31.0, 37.8, 67.6; HRMS (ESI), *m*/*z* calcd for $C_5H_{11}OS$ $[M-H]^-$ 119.0536, found 119.0539.

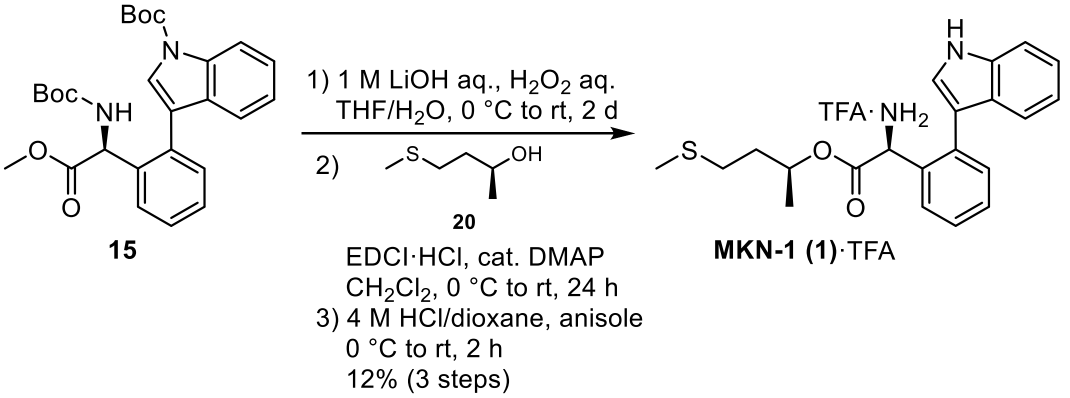

(*S*)-4-(Methylthio) butan-2-yl (*S*)-2-(2-(1*H*-indol-3-yl)phenyl)-2-aminoacetate (1, MKN-1)

To ester **15** (96.1 mg, 0.200 mmol) in THF (2 mL) was added 30% H_2O_2 aq. (91.0 L, 0.800 mmol) at room temperature, then added 1 M LiOH aq. (400 L, 0.400 mmol) at 0 °C. After the reaction mixture was stirred at room temperature for 2 d, the mixture was quenched by saturated aqueous NH_4Cl and extracted with EtOAc, then washed with brine. The combined organic layer was dried over $MgSO_4$, then concentrated under reduced pressure to provide the crude carboxylic acid. To the carboxylic acid in CH_2Cl_2 (1 mL) were added **20** (26.4 mg, 0.220 mmol) in CH_2Cl_2 (1.00 mL), EDCI·HCl (115 mg, 0.600 mmol) and DMAP (9.80 mg, 80.0 mol) at 0 °C. After the reaction mixture was stirred at room temperature for 24 h, quenched by saturated aqueous NH_4Cl and extracted with EtOAc, then washed with brine. The combined organic layer was dried over

$MgSO_4$, then concentrated under reduced pressure to provide the crude ester. To the crude ester were added anisole (87.0 L, 0.800 mmol) and 4 M HCl/dioxane (1.00 mL, 4.00 mmol) at 0 °C. The reaction mixture was stirred at room temperature for 2 h, then concentrated under reduced pressure, followed by purification with HPLC to provide the trifluoroacetate salt of the title compound **1** (11.9 mg, 24.7 mol, 12% in 3 steps) as freeze-dried powder: [ ]$_D$ +115.5 (*c* 0.165, $CHCl_3$); t_R = 20.4 min (linear gradient of B in (A + B), 33 to 40% over 35 min); IR(ATR) ν 1681 (CO) cm^{-1}; ^{1}H-NMR (500 MHz, $CDCl_3$) 1.08 (d, *J* = 6.5 Hz, 3H), 1.59–1.69 (m, 2H), 1.91 (s, 3H), 1.96–2.11 (m, 2H), 4.90–4.94 (m, 1H), 5.35 (br, 1H), 7.08–7.53 (m, 9H), 8.79 (br, 1H); ^{13}C-NMR (125 MHz, $CDCl_3$) 15.5, 19.6, 29.5, 35.0, 53.4, 73.3, 111.7, 113.8, 119.3, 120.3, 122.7, 124.5, 126.8, 127.4, 128.0, 130.0, 130.8, 132.4, 135.9, 136.2, 169.1; HRMS (ESI), *m*/*z* calcd for $C_{21}H_{25}N_2O_2S$ [M+H]$^+$ 369.1631, found 369.1630.

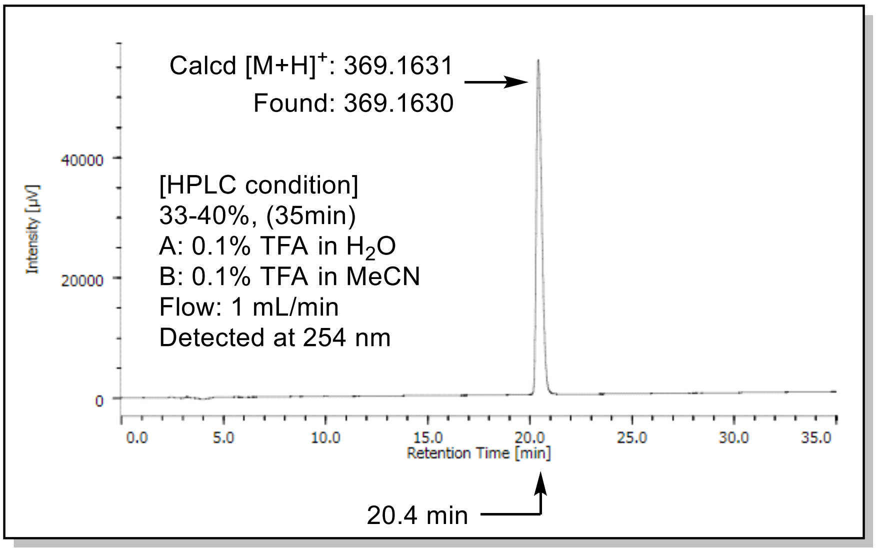

HPLC chart of **MKN-1 (1)**

An MKN-1 derivative, which has a 1-naphthyl group in the place of the indolyl group (22)

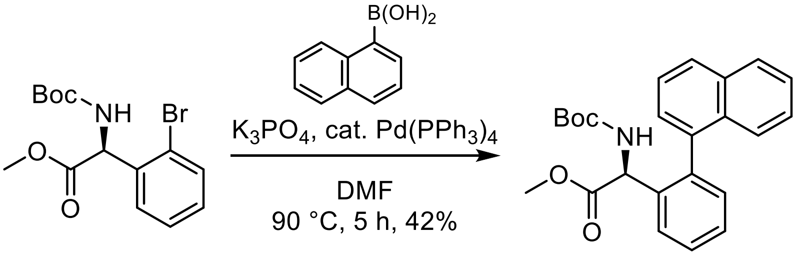

14 **21**

Methyl (*S*)-2-((*tert*-butoxycarbonyl) amino)-2-(2-(naphthalen-1-yl) phenyl)acetate (21)

To a solution of **14** (172 mg, 0.500 mmol) in DMF (10 mL) were added 1-naphthylboronic acid (172 mg, 1.00 mmol), $Pd(PPh_3)_4$ (173 mg, 0.150 mmol) and K_3PO_4 (636 mg, 3.00 mmol) at room

temperature under argon. After stirring at 90 °C for 5 h, the reaction mixture was quenched by saturated aqueous NH_4Cl. The resulting mixture was filtered through a pad of celite, and extracted with EtOAc. The organic layer was dried over $MgSO_4$, then concentrated under reduced pressure, followed by purified by flash column chromatography (PhMe/EtOAc = 30/1) to provide the title compound **21** (83.1 mg, 42% yield) as white solid: [ ]$_D$ +2.79 (c = 0.215, $CHCl_3$); IR(ATR) ν 1708 (CO) cm^{-1}; ^{1}H-NMR (500 MHz, $CDCl_3$) (mixture of diastereomers) 1.24 (s, 9H), 1.36 (s, 9H), 3.93 (s, 3H), 3.46 (s, 3H), 5.03–5.23 (br, 2H), 7.29–7.59 (m, 18H), 7.88–7.91 (m, 4H); ^{13}C-NMR (125 MHz, $CDCl_3$) (mixture of diastereomers) 28.3 (3C), 28.4 (3C), 52.4, 55.1, 55.4, 79.8, 125.2, 125.3, 125.8, 125.9, 126.0, 126.2, 126.6, 127.3, 127.7, 127.8, 128.0, 128.3, 128.4, 128.5, 131.8, 132.5, 132.8, 133.7, 133.8, 135.9, 136.2, 137.4, 137.5, 140.3, 140.5, 154.4, 171.9, 172.0; HRMS (ESI), m/z calcd for $C_{24}H_{25}NNaO_4$ $[M+H]^+$ 414.1676, found 414.1672.

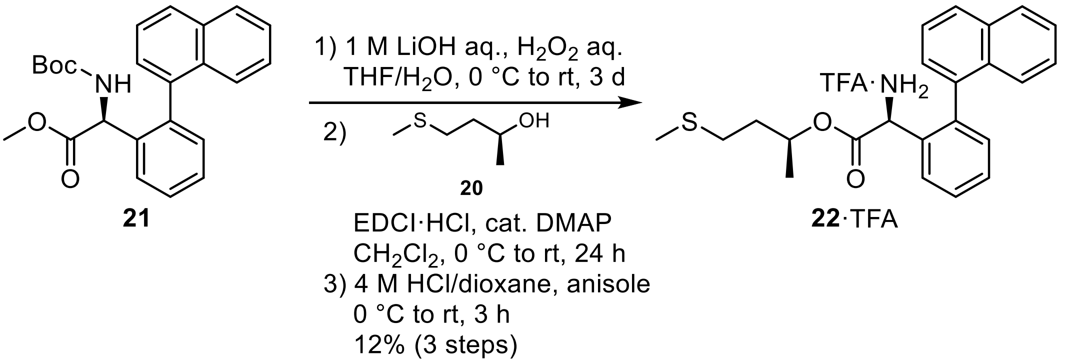

(*S*)-4-(Methylthio) butan-2-yl (*S*)-2-amino-2-(2-(naphthalen-1-yl) phenyl)acetate (22)

To a solution of **21** (39.1 mg, 0.100 mmol) in THF (1 mL) was added 30% H_2O_2 aq. (46.0 L, 0.400 mmol) at room temperature, then was added 1.00 M LiOH aq. (200 L, 0.200 mmol) at 0 °C. After stirring at room temperature for 3 days, the reaction mixture was quenched by saturated aqueous NH_4Cl and extracted with EtOAc, then washed with brine. The combined organic layer was dried over $MgSO_4$, then concentrated under reduced pressure to provide the crude carboxylic acid. To the carboxylic acid in CH_2Cl_2 (0.5 mL) were added **20** (13.2 mg, 0.110 mmol) in CH_2Cl_2 (0.5 mL), EDCI·HCl (67.4 mg, 0.300 mmol) and DMAP (4.90 mg, 400 mol) at 0 °C. After stirring at room temperature for 24 h, the reaction mixture was quenched by saturated aqueous NH_4Cl and extracted with EtOAc, then washed with brine. The combined organic layer was dried over $MgSO_4$, then concentrated under reduced pressure to provide the crude ester. To the crude ester were added anisole (44.0 L, 0.400 mmol) and 4 M HCl/dioxane (0.500 mL, 2.00 mmol) at 0 °C. The reaction mixture was stirred at room temperature for 3 h, then concentrated under reduced pressure, followed by purification with HPLC to provide the trifluoroacetate salt of the title compound **22** (5.90 mg, 12.0 mol, 12% in 3 steps) as freeze-dried powder: [ ]$_D$ +50.1 (c 0.215, MeOH); t_R = 20.4, 21.1, 22.0, 23.6 min (linear gradient of B in (A + B), 36 to 42% over 30 min);

IR(ATR) ν 1673 (CO) cm^{-1}; ^{1}H-NMR (500 MHz, $CDCl_3$) (mixture of diastereomers) 1.04–2.12 (m, 10H), 4.71–4.84 (m, 2H), 7.29–7.50 (m, 9H), 7.91–7.94 (m, 2H); ^{13}C-NMR (125 MHz, $CDCl_3$) (mixture of diastereomers) 15.4, 15.6, 19.4, 19.6, 29.4, 29.6, 29.9, 34.7, 35.1, 35.2, 53.4, 53.5, 53.6, 73.4, 73.6, 73.7, 125.1, 125.4, 125.8, 126.2, 126.6, 126.9, 127.4, 127.8, 127.9, 128.2, 128.5, 128.6, 128.9, 129.0, 129.1, 129.2, 129.9, 130.0 131.0, 131.2, 131.9, 132.0, 132.1, 132.3, 132.4, 133.8, 133.9, 136.0, 136.1, 140.9, 141.3, 141.4, 168.4, 168.8, 169.1, 169.3; HRMS (ESI), *m*/*z* calcd for $C_{23}H_{26}NO_2S$ $[M+H]^+$ 380.1679, found 380.1675.

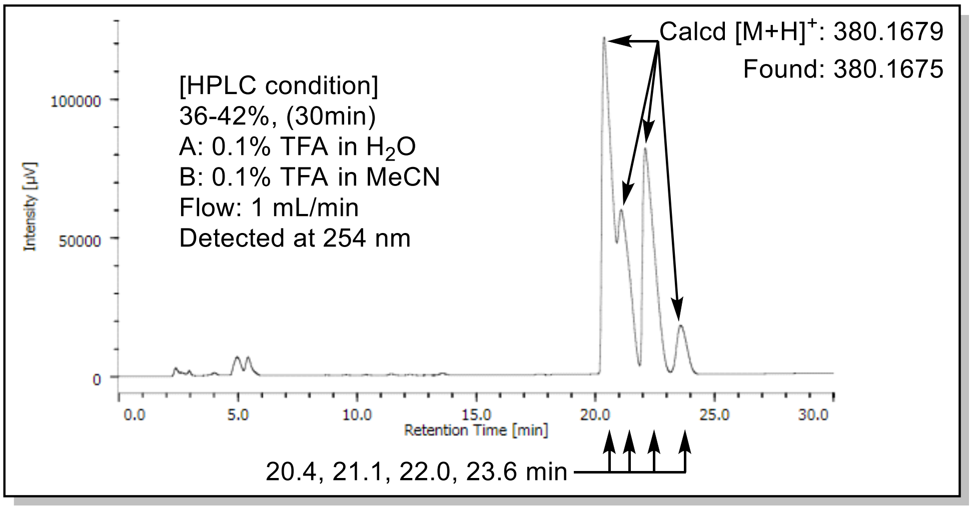

HPLC chart of compound **22**

An MKN-1 derivative, which has a 2-naphthyl group in the place of the indolyl group (24)

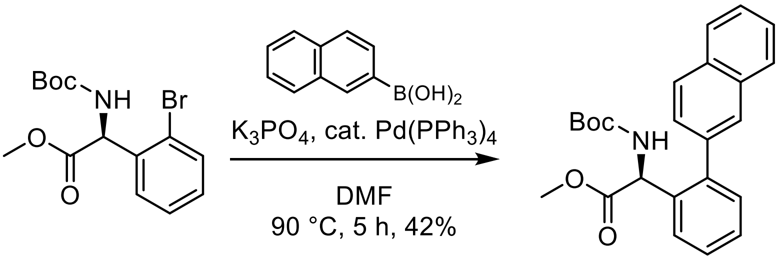

14 **23**

Methyl (*S*)-2-((*tert*-butoxycarbonyl) amino)-2-(2-(naphthalen-2-yl) phenyl)acetate (23)

To a solution of **14** (172 mg, 0.500 mmol) in DMF (10 mL) were added 2-naphthylboronic acid (172 mg, 1.00 mmol), Pd $(PPh_3)_4$ (173 mg, 0.150 mmol) and K_3PO_4 (636 mg, 3.00 mmol) at room temperature under argon. After stirring at 90 °C for 5 h, the reaction mixture was quenched by saturated aqueous NH_4Cl. The resulting mixture was filtered through a pad of celite, and extracted with EtOAc. The organic layer was dried over $MgSO_4$, then concentrated under reduced pressure, followed by purified by flash column chromatography (PhMe/EtOAc = 30/1) to provide the title compound **23** (81.6 mg, 42% yield) as white solid: [ ]$_D$ +0.31 (*c* = 0.64, $CHCl_3$); IR(ATR) ν

1709 (CO) cm^{-1}; ^{1}H-NMR (500 MHz, $CDCl_3$) 1.39 (s, 9H), 3.64 (s, 3H), 5.32–5.49 (m, 1H), 7.38–7.60 (m, 7H), 7.85–7.93 (m, 4H); ^{13}C-NMR (125 MHz, $CDCl_3$) 28.4 (3C), 52.7, 54.7, 80.1, 126.3, 126.4, 126.9, 127.7, 127.9, 128.0, 128.3, 128.6, 131.3, 132.7, 133.3, 134.7, 137.8, 142.5, 154.6, 172.3; HRMS (ESI), *m*/*z* calcd for $C_{24}H_{25}NNaO_4$ $[M+H]^+$ 414.1676, found 414.1674.

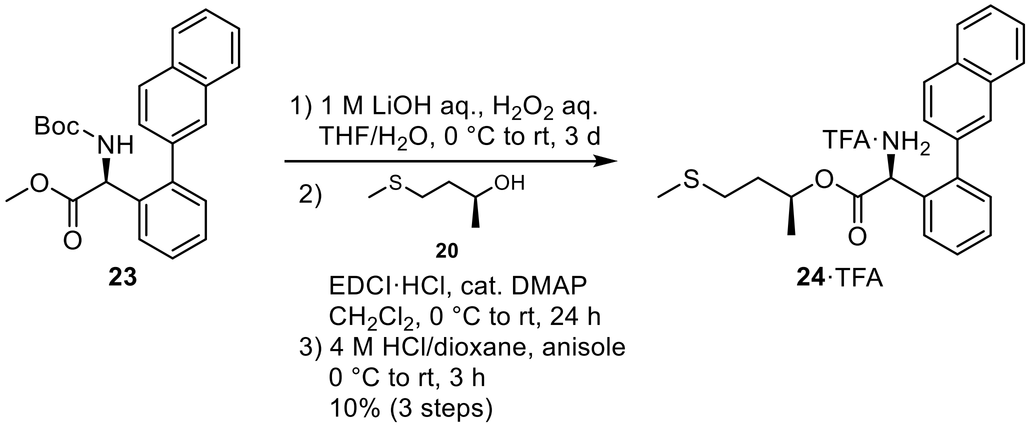

(*S*)-4-(Methylthio) butan-2-yl (*S*)-2-amino-2-(2-(naphthalen-2-yl)phenyl)acetate (24)

To a solution of **23** (39.1 mg, 0.100 mmol) in THF (1 mL) was added 30% H_2O_2 aq. (46.0 L, 0.400 mmol) at room temperature, then was added 1.00 M LiOH aq. (200 L, 0.200 mmol) at 0 °C. After stirring at room temperature for 3 days, the reaction mixture was quenched by saturated aqueous NH_4Cl and extracted with EtOAc, then washed with brine. The combined organic layer was dried over $MgSO_4$, then concentrated under reduced pressure to provide the crude carboxylic acid. The carboxylic acid in CH_2Cl_2 (0.5 mL) were added **20** (13.2 mg, 0.110 mmol) in CH_2Cl_2 (0.5 mL), EDCI·HCl (67.4 mg, 0.300 mmol) and DMAP (4.90 mg, 400 mol) at 0 °C. After stirring at room temperature for 24 h, the reaction mixture was quenched by saturated aqueous NH_4Cl and extracted with EtOAc, then washed with brine. The combined organic layer was dried over $MgSO_4$, then concentrated under reduced pressure to provide the crude ester. To the crude ester were added anisole (44.0 L, 0.400 mmol) and 4 M HCl/dioxane (0.500 mL, 2.00 mmol) at 0 °C. The reaction mixture was stirred at room temperature for 3 h, then concentrated under reduced pressure, followed by purification with HPLC to provide the trifluoroacetate salt of the title compound **24** (5.10 mg, 10.3 mol, 10% in 3 steps) as freeze-dried powder: [ ]$_D$ +136.2 (*c* 0.20, MeOH); t_R = 25.3 min (linear gradient of B in (A + B), 36 to 42% over 30 min); IR(ATR) ν 1673 (CO) cm^{-1}; ^{1}H-NMR (500 MHz, $CDCl_3$) 1.10 (d, *J* = 6.0 Hz, 3H), 1.55–1.73 (m, 2H), 1.89 (s, 3H), 1.94–1.98 (m, 2H), 4.77 (br s, 1H), 5.15 (br s, 1H), 7.39–7.52 (m, 7H), 7.85–7.90 (m, 4H); ^{13}C-NMR (125 MHz, $CDCl_3$) 15.6, 19.6, 29.4, 35.2, 53.2, 73.5, 126.5, 126.6, 126.8, 127.3, 127.9, 128.3, 128.6, 128.7, 128.8, 129.8, 130.0, 131.5, 132.8, 133.3, 136.6, 143.1, 168.6; HRMS (ESI), *m*/*z* calcd for $C_{23}H_{26}NO_2S$ $[M+H]^+$ 380.1679, found 380.1676.

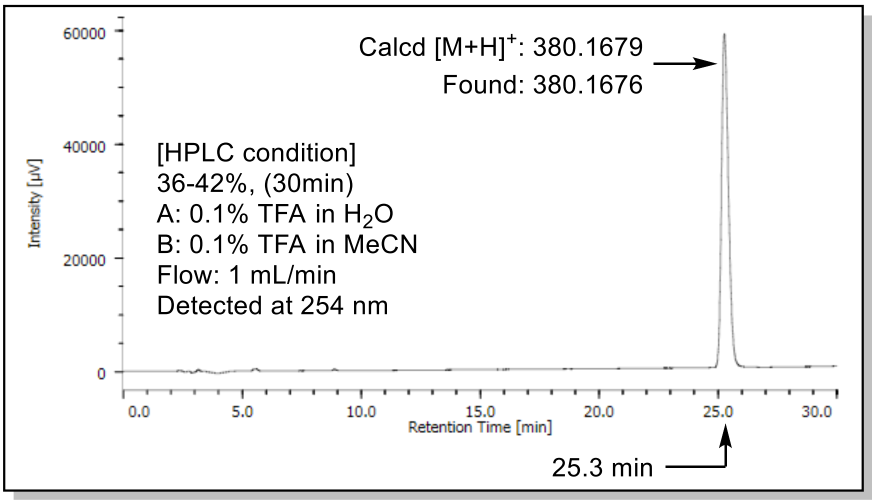

HPLC chart of compound **24**

An MKN-1 derivative, which has a benzofuranyl group in the place of the indolyl group (27)

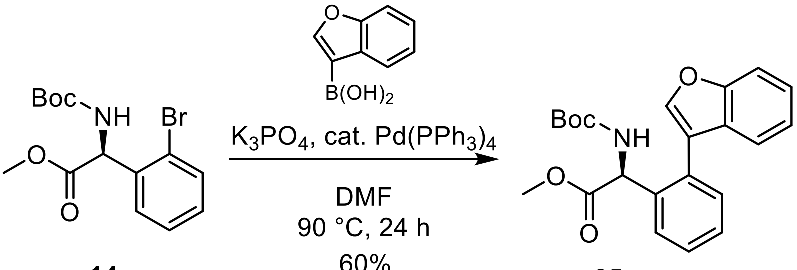

14 **25**

Methyl (*S*)-2-(2-(benzofuran-3-yl) phenyl)-2-((*tert*-butoxycarbonyl)amino)acetate (25)

To a solution of **14** (172 mg, 0.500 mmol) in DMF (10 mL) were added benzofuran-3-boronic acid (162 mg, 1.00 mmol), Pd $(PPh_3)_4$ (173 mg, 0.150 mmol) and K_3PO_4 (636 mg, 3.00 mmol) at room temperature under argon. After stirring at 90 °C for 24 h, the reaction mixture was quenched by saturated aqueous NH_4Cl. The resulting mixture was filtered through a pad of celite, and extracted with EtOAc. The organic layer was dried over $MgSO_4$, then concentrated under reduced pressure, followed by purified by flash column chromatography (PhMe/EtOAc = 30/1) to provide the title compound **25** (114 mg, 60% yield) as yellow oil:[ ]$_D$ -14.5 (*c* = 1.02, $CHCl_3$); IR(ATR) ν 1744 (CO), 1709 (CO) cm^{-1}; ^{1}H-NMR (500 MHz, $CDCl_3$) 1.39 (s, 9H), 3.59 (s, 3H), 5.35–5.58 (m, 1H), 7.24–7.58 (m, 8H), 7.87 (s, 1H); ^{13}C-NMR (125 MHz, $CDCl_3$) 28.4 (3C), 52.7, 54.8, 80.2, 111.8 (2C), 119.4, 120.4, 123.0, 124.8, 127.4, 128.3, 128.6, 128.7, 131.5, 136.0, 143.2, 155.4, 172.1; HRMS (ESI), *m*/*z* calcd for $C_{22}H_{24}NO_5$ $[M+H]^+$ 382.2649, found 382.2644.

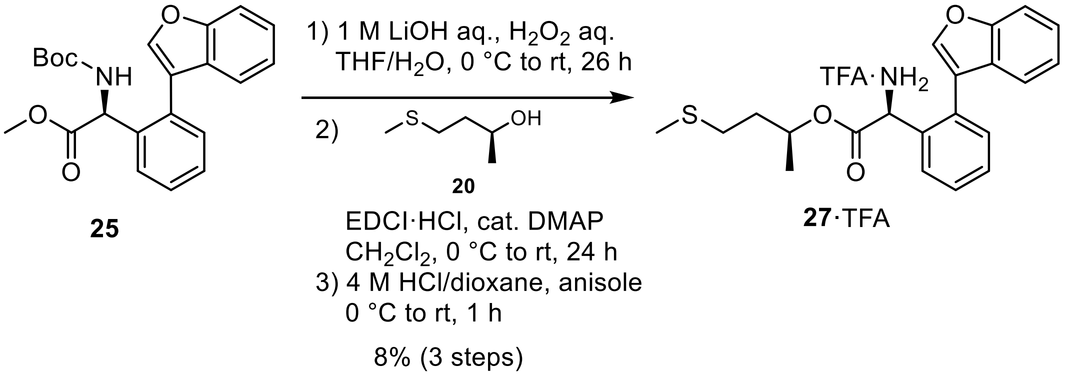

(*S*)-4-(Methylthio) butan-2-yl (*S*)-2-amino-2-(2-(benzofuran-3-yl)phenyl)acetate (27)

To a solution of **25** (275 mg, 0.721 mmol) in THF (7.21 mL) was added 30% H_2O_2 aq. (327 L, 2.88 mmol) at room temperature, then was added 1.00 M LiOH aq. (1.44 mL, 1.44 mmol) at 0 °C. After stirring at room temperature for 26 h, the reaction mixture was quenched by saturated aqueous NH_4Cl and extracted with EtOAc, then washed with brine. The combined organic layer was dried over $MgSO_4$, then concentrated under reduced pressure to provide the crude carboxylic acid. To the carboxylic acid in CH_2Cl_2 (3.6 mL) were added **20** (95.2 mg, 0.793 mmol) in CH_2Cl_2 (3.6 mL), EDCI·HCl (413 mg, 2.16 mmol) and DMAP (35.2 mg, 0.288 mmol) at 0 °C. After stirring at room temperature for 24 h, the reaction mixture was quenched by saturated aqueous NH_4Cl and extracted with EtOAc, then washed with brine. The combined organic layer was dried over $MgSO_4$, then concentrated under reduced pressure to provide the crude ester. To the crude ester were added anisole (313 L, 2.88 mmol) and 4 M HCl/dioxane (3.61 mL, 14.4 mmol) at 0 °C. The reaction mixture was stirred at room temperature for 1 h, then concentrated under reduced pressure, followed by purification with HPLC to provide the trifluoroacetate salt of the title compound **27** (27.1 mg, 56.1 mol, 8% in 3 steps) as freeze-dried powder: [ ]$_D$ +92.8 (*c* 0.095, MeOH); t_R = 23.8 min (linear gradient of B in (A + B), 30 to 50% over 50 min); IR(ATR) ν 1674 (CO) cm^{-1}; ^{1}H-NMR (500 MHz, $CDCl_3$) 1.06 (d, *J* = 6.0 Hz, 3H), 1.55–1.68 (m, 2H), 1.90 (s, 3H), 1.98 (br, 2H), 4.79–4.85 (m, 1H), 5.18 (br, 1H), 7.23–7.55 (m, 8H), 7.53 (s, 1H); ^{13}C-NMR (125 MHz, $CDCl_3$) 15.5, 19.6, 29.4, 35.2, 53.4, 73.2, 111.8, 118.8, 120.2, 123.2, 125.0, 126.9, 128.0, 129.2, 129.9, 131.7, 131.9 (2C), 143.6, 155.3, 168.8; HRMS (ESI), *m*/*z* calcd for $C_{21}H_{24}N_2O_3S$ $[M+H]^+$ 370.1471, found 370.1472.

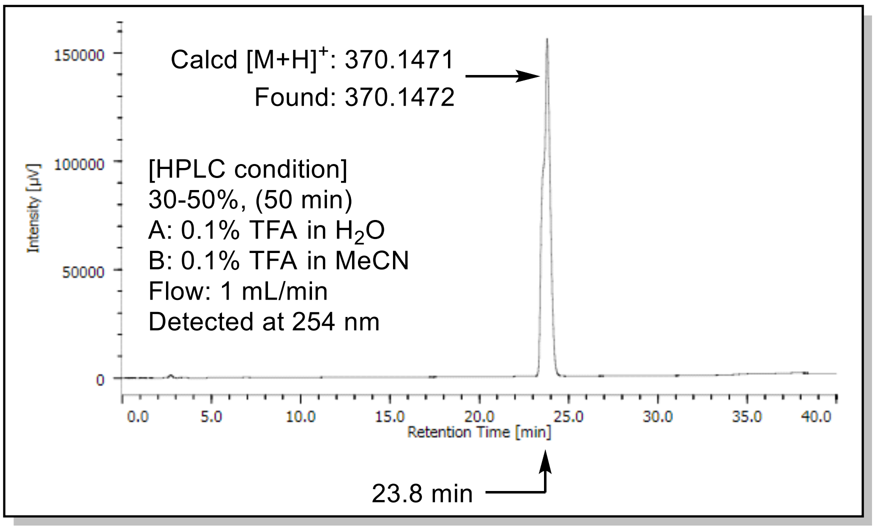

HPLC chart of compound **27**

An MKN-1 derivative, which has a benzothiophenyl group in the place of the indolyl group (28)

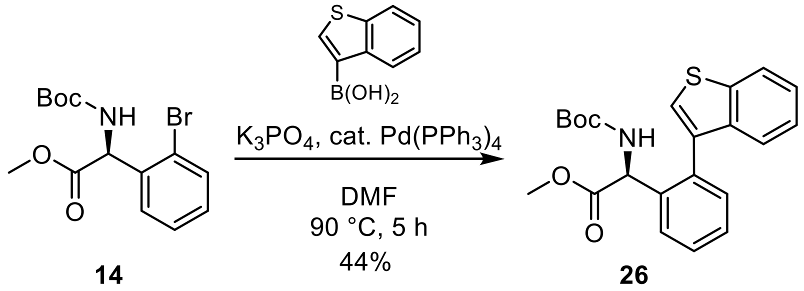

Methyl (*S*)-2-(2-(benzo[*b*]thiophen-3-yl) phenyl)-2-((*tert*-butoxycarbonyl) amino)acetate (26)

To a solution of **14** (688 mg, 2.00 mmol) in DMF (10 mL) were added Benzo[*b*]thiophene-3-boronic acid (712 mg, 4.00 mmol), Pd $(PPh_3)_4$ (692 mg, 0.600 mmol) and K_3PO_4 (2.54 g, 12.0 mmol) at room temperature under argon. After stirring at 90 °C for 5 h, the reaction mixture was quenched by saturated aqueous NH_4Cl. The resulting mixture was filtered through a pad of celite, and extracted with EtOAc. The organic layer was dried over $MgSO_4$, then concentrated under reduced pressure, followed by purified by flash column chromatography (PhMe/EtOAc = 40/1) to provide the title compound **26** (347 mg, 44% yield) as brown oil: [ ]$_D$ -0.36 (*c* = 1.03, $CHCl_3$); IR(ATR) ν 1708 (CO) cm^{-1}; ^{1}H-NMR (500 MHz, $CDCl_3$) 1.37 (s, 9H), 3.46 (s, 3H), 5.19–5.46 (m, 1H), 7.31–7.55 (m, 8H), 7.92 (d, *J* = 8.0 Hz, 1H); ^{13}C-NMR (125 MHz, $CDCl_3$) 28.4 (3C), 52.6, 54.4, 80.0, 122.8 (2C), 123.2, 124.2, 124.5, 125.2, 125.9, 127.2, 128.5, 128.7, 131.5, 135.0, 135.7, 140.2, 154.6, 171.9; HRMS (ESI), *m/z* calcd for $C_{22}H_{24}NO_4S$ [M+H]$^+$ 398.1421, found 398.1417.

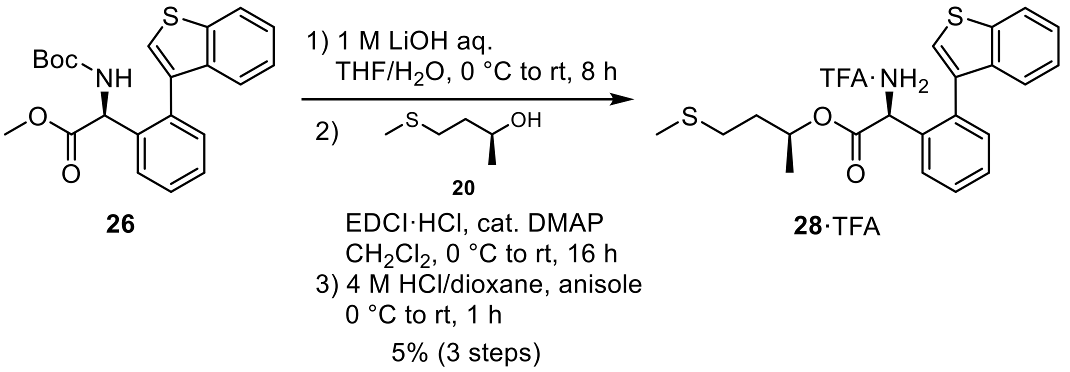

(*S*)-4-(Methylthio) butan-2-yl (*S*)-2-amino-2-(2-(benzo[*b*]thiophen-3-yl) phenyl)acetate (28)

To a solution of **26** (252 mg, 0.633 mmol) in THF (6.3 mL) was added 1.00 M LiOH aq. (1.27 mL, 1.27 mmol) at 0 °C. After stirring at room temperature for 8 h, the reaction mixture was quenched by saturated aqueous NH_4Cl and extracted with EtOAc, then washed with brine. The combined organic layer was dried over $MgSO_4$, then concentrated under reduced pressure to provide the crude carboxylic acid. To the carboxylic acid in CH_2Cl_2 (3.2 mL) were added **20** (85.6 mg, 0.700 mmol) in CH_2Cl_2 (3.2 mL), EDCI·HCl (363 mg, 1.90 mmol) and DMAP (30.9 mg, 0.253 mmol) at 0 °C. After stirring at room temperature for 16 h, the reaction mixture was quenched by saturated aqueous NH_4Cl and extracted with EtOAc, then washed with brine. The combined organic layer was dried over $MgSO_4$, then concentrated under reduced pressure to provide the crude ester. To the crude ester were added anisole (275 L, 2.53 mmol) and 4 M HCl/dioxane (3.17 mL, 12.7 mmol) at 0 °C. The reaction mixture was stirred at room temperature for 1 h, then concentrated under reduced pressure, followed by purification with HPLC to provide the trifluoroacetate salt of the title compound **28** (17.2 mg, 34.4 mol, 5% in 3 steps) as freeze-dried powder: [ ]$_D$ +78.7 (*c* 0.095, MeOH); t_R = 26.5 min (linear gradient of B in (A + B), 30 to 50% over 40 min); IR(ATR) ν 1673 (CO) cm^{-1}; ^{1}H-NMR (500 MHz, $CDCl_3$) 0.96 (d, *J* = 6.0 Hz, 3H), 1.60–1.63 (m, 2H), 1.95 (s, 3H), 2.01–2.06 (m, 2H), 4.81 (br, 1H), 4.95 (br, 1H), 7.30–7.52 (m, 7H), 7.72 (s, 1H), 7.92 (d, *J* = 8.0 Hz, 1H); ^{13}C-NMR (125 MHz, $CDCl_3$) 15.6, 19.5, 29.5, 35.1, 53.5, 73.4, 122.9 (2C), 124.3, 124.8, 125.1, 126.8, 127.0, 129.4, 130.2, 131.9, 133.6 136.8, 139.5, 140.4, 168.5; HRMS (ESI), *m*/*z* calcd for $C_{21}H_{24}N_2O_2S_2$ $[M+H]^+$ 386.1243, found 386.1239.

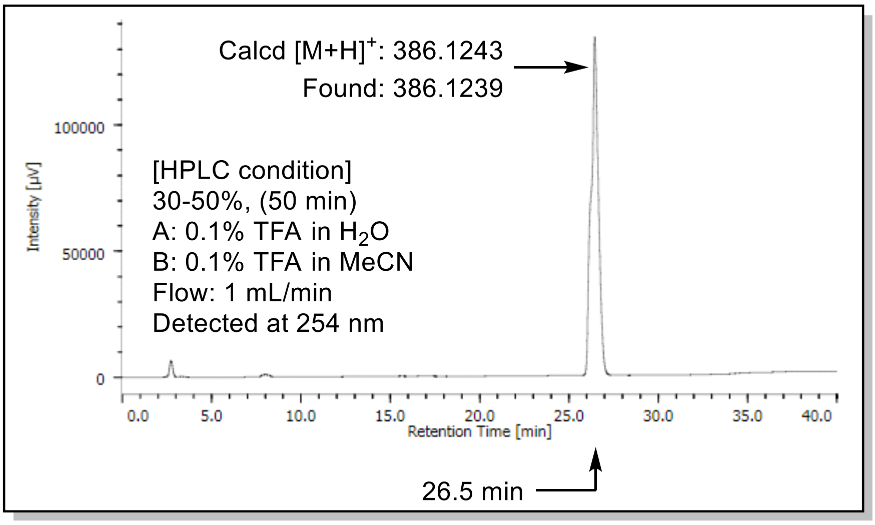

HPLC chart of compound **28**

An MKN-1 derivative, which has a methoxy group in the place of the methanesulfidyl group (30)

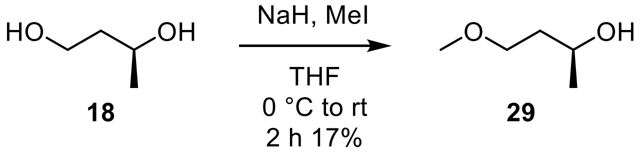

(*S*)-4-Methoxybutan-2-ol (29)

To a solution of (*S*)-(+)-1,3-butanediol (**18**) (268   L, 3.00 mmol) in THF (15.0 mL) was added NaH (144 mg, 3.60 mmol) at 0 °C under argon. The reaction mixture was stirred at room temperature for 30 min, and MeI (224   L, 3.60 mmol) was then added at room temperature. After stirring at room temperature for 17 h, the reaction mixture was quenched by saturated aqueous NH_4Cl and extracted with $CHCl_3$. The organic layer was dried over $MgSO_4$, then concentrated under reduced pressure, followed by purified by flash column chromatography (MeOH/$CHCl_3$ = 1/49 to 1/9) to provide the title compound **29** (52.9 mg, 17% yield) as colorless oil: [ ]$_D$ +4.29 (*c* 0.07, $CHCl_3$); IR(ATR) ν 3406 (OH) cm^{-1}; ^{1}H-NMR (500 MHz, $CDCl_3$)   1.20 (d, *J* = 6.5 Hz, 3H), 1.65–1.77 (m, 2H), 3.36 (s, 3H), 3.53–3.64 (m, 2H), 3.96–4.02 (m, 1H); ^{13}C-NMR (125 MHz, $CDCl_3$)   23.5, 38.2, 59.1, 67.9, 72.0; HRMS (ESI), *m*/*z* calcd for $C_5H_{12}NaO_2$ [M+Na]$^+$ 127.0730, found 127.0730.

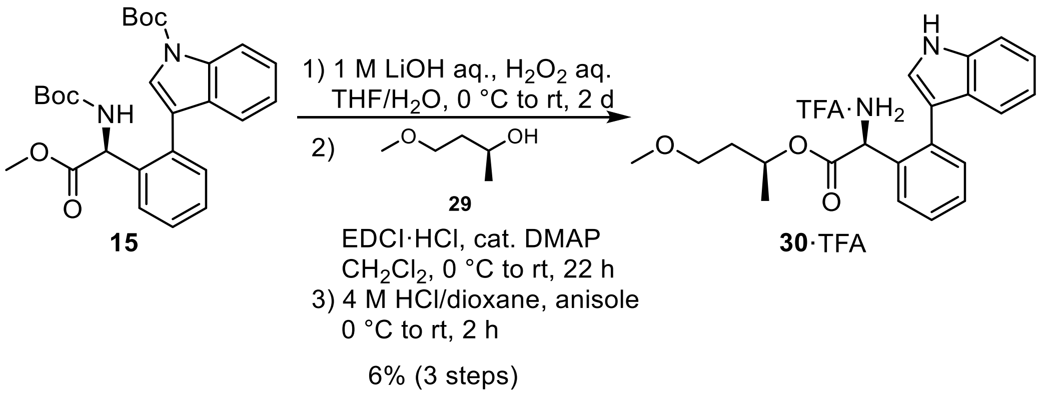

(*S*)-4-Methoxybutan-2-yl (*S*)-2-(2-(1*H*-indol-3-yl) phenyl)-2-aminoacetate (30)

To a solution of **15** (96.1 mg, 0.200 mmol) in THF (2 mL) was added 30% H_2O_2 aq. (91.0 L, 0.800 mmol) at room temperature, then added a 1.00 M LiOH aq. (400 L, 0.400 mmol) at 0 °C. After stirring at room temperature for 2 days, the reaction mixture was quenched by saturated aqueous NH_4Cl and extracted with EtOAc, then washed with brine. The combined organic layer was dried over $MgSO_4$, then concentrated under reduced pressure to provide the crude carboxylic acid. To the carboxylic acid in CH_2Cl_2 (1 mL) were added **29** (22.9 mg, 0.220 mmol) in CH_2Cl_2 (1 mL), EDCI·HCl (115 mg, 0.600 mmol) and DMAP (9.80 mg, 80.0 mol) at 0 °C. After stirring at room temperature for 22 h, the reaction mixture was quenched by saturated aqueous NH_4Cl and extracted with EtOAc, then washed with brine. The combined organic layer was dried over $MgSO_4$, then concentrated under reduced pressure to provide the crude ester. To the crude ester were added anisole (87.0 L, 0.800 mmol) and 4 M HCl/dioxane (1.00 mL, 4.00 mmol) at 0 °C. The reaction mixture was stirred at room temperature for 2 h, then concentrated under reduced pressure, followed by purification with HPLC to provide the trifluoroacetate salt of the title compound **30** (5.20 mg, 11.1 mol, 6% in 3 steps) as freeze-dried powder: [ ]$_D$ +95.9 (*c* 0.20, MeOH); t_R = 20.7 min (linear gradient of B in (A + B), 28 to 34% over 30 min); IR(ATR) ν 1672 (CO) cm^{-1}; ^{1}H-NMR (500 MHz, $CDCl_3$) 1.05 (d, *J* = 2.0 Hz, 3H), 1.62 (br, 2H), 2.93–3.08 (m, 2H), 3.11 (s, 3H), 4.94 (br, 1H), 5.43 (br, 1H), 7.07–7.54 (m, 9H), 9.96 (br, 1H); ^{13}C-NMR (125 MHz, $CDCl_3$) 19.6, 35.4, 53.6, 58.4, 68.1, 71.9, 111.6, 113.5, 119.0, 120.1, 122.5, 124.4, 126.6, 127.2, 127.9, 129.9, 130.4, 132.3, 135.9, 136.1, 168.8; HRMS (ESI), *m*/*z* calcd for $C_{21}H_{25}N_2O_3$ $[M+H]^+$ 353.1860, found 353.1864.

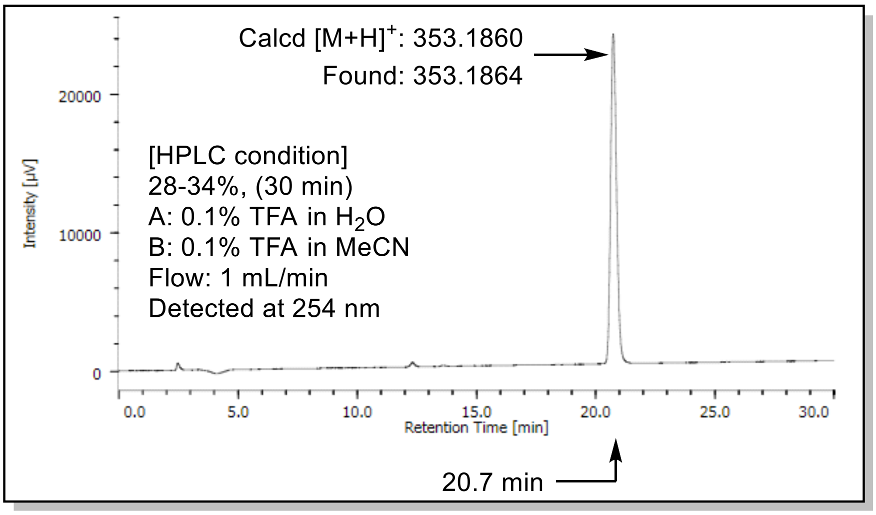

HPLC chart of compound **30**

An MKN-1 derivative, which has a *tert*-butyl sulfidyl group in the place of the methanesulfidyl group (34)

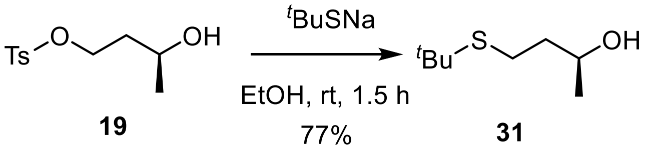

(*S*)-4-(*tert*-Butylthio) butan-2-ol (31)

Compound **19** was synthesized using the previously reported procedure.[70] To a solution of **19** (158 mg, 0.600 mmol) in EtOH (3 mL) was added ^{t}BuSNa (89.7 mg, 0.720 mmol) at room temperature under argon. After stirring at room temperature for 1.5 h, the reaction mixture was quenched with H_2O and extracted with EtOAc. The organic layer was dried over $MgSO_4$, then concentrated under reduced pressure, followed by purified by flash column chromatography (hexane/EtOAc = 3/1) to provide the title compound **31** (74.7 mg, 77% yield) as colorless oil: [ ]$_D$ +8.9 (*c* 0.315, $CHCl_3$); IR(ATR) ν 3389 (OH) cm^{-1}; ^{1}H-NMR (400 MHz, $CDCl_3$) 1.21 (d, *J* = 6.0 Hz, 3H), 1.33 (s, 9H), 1.69–1.74 (m, 2H), 2.65 (t, *J* = 6.0 Hz, 2H), 3.90–3.98 (m, 1H); ^{13}C-NMR (125 MHz, $CDCl_3$) 23.7, 25.1, 31.1 (3C), 38.7, 42.3, 67.7; HRMS (ESI), *m*/*z* calcd for $C_8H_{18}NaOS$ [M+Na]$^+$ 185.0971, found 185.0975.

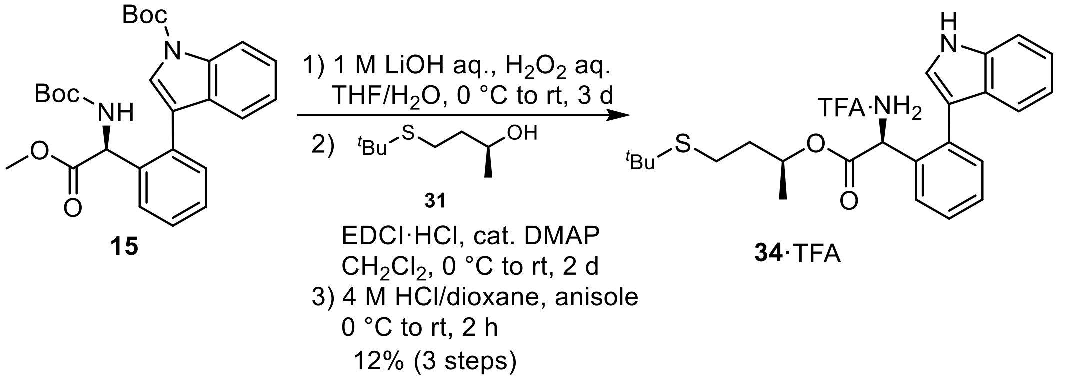

(*S*)-4-(*tert*-Butylthio) butan-2-yl (*S*)-2-(2-(1*H*-indol-3-yl)phenyl)-2-aminoacetate (34)

To a solution of **15** (144 mg, 0.300 mmol) in THF (3 mL) was added 30% H_2O_2 aq. (136 L, 1.20 mmol) at room temperature, then added a 1.00 M LiOH aq. (600 L, 0.600 mmol) at 0 °C. After stirring at room temperature for 3 days, the reaction mixture was quenched by saturated aqueous NH_4Cl and extracted with EtOAc, then washed with brine. The combined organic layer was dried over $MgSO_4$, then concentrated under reduced pressure to provide the crude carboxylic acid. To the carboxylic acid in CH_2Cl_2 (1.5 mL) were added **31** (48.7 mg, 0.300 mmol) in CH_2Cl_2 (1.5 mL), EDCI·HCl (172 mg, 0.900 mmol) and DMAP (14.7 mg, 0.120 mmol) at 0 °C. After stirring at room temperature for 2 days, the reaction mixture was quenched by saturated aqueous NH_4Cl and extracted with EtOAc, then washed with brine. The combined organic layer was dried over $MgSO_4$, then concentrated under reduced pressure to provide the crude ester. To the crude ester were added anisole (130 L, 1.20 mmol) and 4 M HCl/dioxane (1.50 mL, 6.00 mmol) at 0 °C. The reaction mixture was stirred at room temperature for 2 h, then concentrated under reduced pressure, followed by purification with HPLC to provide the trifluoroacetate salt of the title compound **34** (18.3 mg, 34.9 mol, 12% in 3 steps) as freeze-dried powder: [ ]$_D$ +106.9 (*c* 0.035, $CHCl_3$); t_R = 26.5 min (linear gradient of B in (A + B), 35 to 50% over 45 min); IR(ATR) ν 1679 (CO) cm^{-1}; ^{1}H-NMR (500 MHz, $CDCl_3$) 1.09 (d, *J* = 6.0 Hz, 3H), 1.17 (s, 9H), 1.51–1.73 (m, 2H), 2.12 (br, 3H), 4.89 (br, 1H), 5.37 (br, 1H), 7.33–7.51 (m, 9H), 8.87 (br, 1H); ^{13}C-NMR (125 MHz, $CDCl_3$) 19.7, 23.8, 31.0 (3C), 36.0, 42.3, 53.6, 74.1, 111.8, 113.7, 119.3, 120.3, 122.7, 124.5, 126.7, 127.4, 128.2, 130.1, 130.6, 132.5, 136.0, 136.2, 169.1; HRMS (ESI), *m*/*z* calcd for $C_{24}H_{31}N_2O_2S$ $[M+H]^+$ 411.2101, found 411.2105.

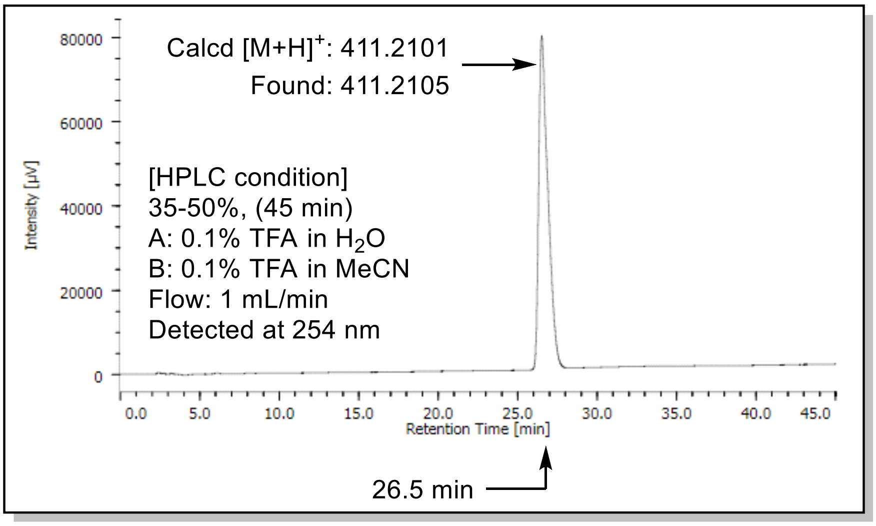

HPLC chart of compound **34**

An MKN-1 derivative, which has an *iso*-propyl sulfidyl group in the place of the methanesulfidyl group (35)

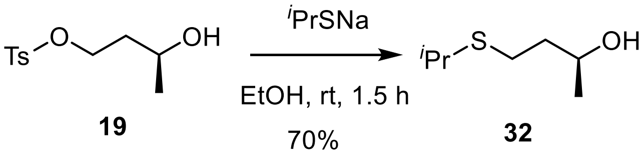

(*S*)-4-(Isopropylthio) butan-2-ol (32)

Compound **19** was synthesized using the previously reported procedure.[70] To a solution of **19** (158 mg, 0.600 mmol) in EtOH (3 mL) was added ^{i}PrSNa (78.5 mg, 0.720 mmol) at room temperature under argon. After stirring at room temperature for 1.5 h, the reaction mixture was quenched with H_2O and extracted with EtOAc. The organic layer was dried over $MgSO_4$, then concentrated under reduced pressure, followed by purified by flash column chromatography (hexane/EtOAc = 3/1) to provide the title compound **32** (62.3 mg, 70% yield) as colorless oil: [ ]$_D$ +7.5 (*c* 0.265, $CHCl_3$); IR(ATR) ν 3392 (OH) cm^{-1}; ^{1}H-NMR (500 MHz, $CDCl_3$) 1.22 (d, *J* = 6.0 Hz, 3H), 1.27 (d, *J* = 6.0 Hz, 6H), 1.70–1.74 (m, 2H), 2.642–2.67 (m, 2H), 2.91–3.00 (m, 1H), 3.92–3.98 (m, 1H); ^{13}C-NMR (125 MHz, $CDCl_3$) 23.5 (2C), 23.7, 27.3, 35.0, 38.5, 67.7; HRMS (ESI), *m*/*z* calcd for $C_7H_{16}NaOS$ [M+Na]$^+$ 171.0814, found 171.0816.

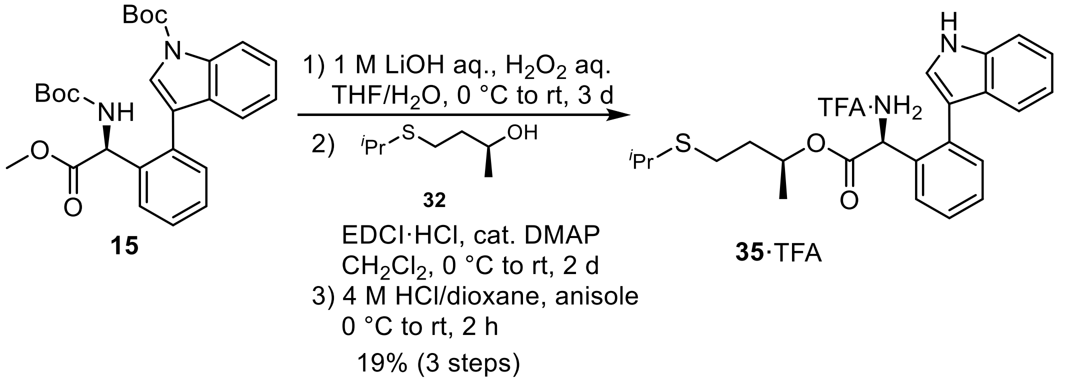

(*S*)-4-(Isopropylthio) butan-2-yl (*S*)-2-(2-(1*H*-indol-3-yl)phenyl)-2-aminoacetate (35)

To a solution of **15** (144 mg, 0.300 mmol) in THF (3 mL) was added 30% H_2O_2 aq. (136 L, 1.20 mmol) at room temperature, then added 1.00 M LiOH aq. (600 L, 0.600 mmol) at 0 °C. After stirring at room temperature for 3 days, the reaction mixture was quenched by saturated aqueous NH_4Cl and extracted with EtOAc, then washed with brine. The combined organic layer was dried over $MgSO_4$, then concentrated under reduced pressure to provide the crude carboxylic acid. To the carboxylic acid in CH_2Cl_2 (1.5 mL) were added **32** (44.5 mg, 0.300 mmol) in CH_2Cl_2 (1.5 mL), EDCI·HCl (172 mg, 0.900 mmol) and DMAP (14.7 mg, 0.120 mmol) at 0 °C. After stirring at room temperature for 2 days, the reaction mixture was quenched by saturated aqueous NH_4Cl and extracted with EtOAc, then washed with brine. The combined organic layer was dried over $MgSO_4$, then concentrated under reduced pressure to provide the crude ester. To the crude ester were added anisole (130 L, 1.20 mmol) and 4 M HCl/dioxane (1.50 mL, 6.00 mmol) at 0 °C. The reaction mixture was stirred at room temperature for 2 h, then concentrated under reduced pressure, followed by purification with HPLC to provide the trifluoroacetate salt of the title compound **35** (26.2 mg, 56.4 mol, 19% in 3 steps) as freeze-dried powder: $[\alpha]_D$ +65.4 (*c* 0.095, $CHCl_3$); t_R = 21.8 min (linear gradient of B in (A + B), 35 to 50% over 45 min); IR(ATR) ν 1683 (CO) cm^{-1}; ^1H-NMR (500 MHz, $CDCl_3$) δ 1.12–1.14 (m, 9H), 1.58–1.74 (m, 2H), 2.05–2.19 (m, 2H), 2.68–2.73 (m, 1H), 4.96 (br, 1H), 5.40 (br, 1H), 7.08–7.53 (m, 9H), 8.81 (br, 1H); ^{13}C-NMR (125 MHz, $CDCl_3$) δ 19.7, 23.4 (2C), 25.9, 35.0, 35.7, 53.6, 73.8 111.8, 113.8, 119.3, 120.4, 122.8, 124.5, 126.8, 127.4, 128.2, 130.1, 130.6, 132.5, 135.9, 136.2, 169.0; HRMS (ESI), *m*/*z* calcd for $C_{23}H_{29}N_2O_2S$ $[M+H]^+$ 397.1944, found 397.1941.

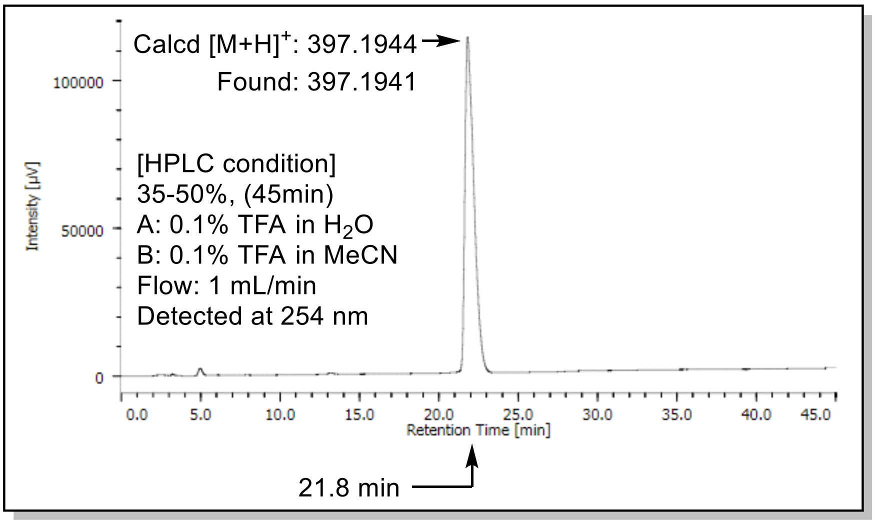

HPLC chart of compound **35**

An MKN-1 derivative, which has a benzenesulfidyl group in the place of the methanesulfidyl group (36)

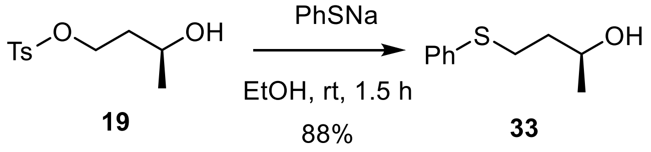

(*S*)-4-(Phenylthio) butan-2-ol (33)

Compound **19** was synthesized using the previously reported procedure.[70] To a solution of **19** (158 mg, 0.600 mmol) in EtOH (3 mL) was added PhSNa (106 mg, 0.720 mmol) at room temperature under argon. After stirring at room temperature for 1.5 h, the reaction mixture was quenched with H_2O and extracted with EtOAc. The organic layer was dried over $MgSO_4$, then concentrated under reduced pressure, followed by purified by flash column chromatography (hexane/EtOAc = 3/1) to provide the title compound **33** (96.1 mg, 88% yield) as white solid: [ ]$_D$ +31.1 (*c* 0.260, $CHCl_3$); IR(ATR) ν 3359 (OH) cm^{-1}; ^{1}H-NMR (500 MHz, $CDCl_3$) 1.22 (d, *J* = 6.5 Hz, 3H), 1.75–1.80 (m, 2H), 2.99–3.10 (m, 2H), 3.95–4.01 (m, 1H), 7.16–7.36 (m, 5H); ^{13}C-NMR (125 MHz, $CDCl_3$) 23.6, 30.1, 38.1, 67.0, 125.9, 128.9 (2C), 129.1 (2C), 136.3; HRMS (ESI), *m*/*z* calcd for $C_{10}H_{14}NaOS$ [M+Na]$^+$ 205.0658, found 205.0662.

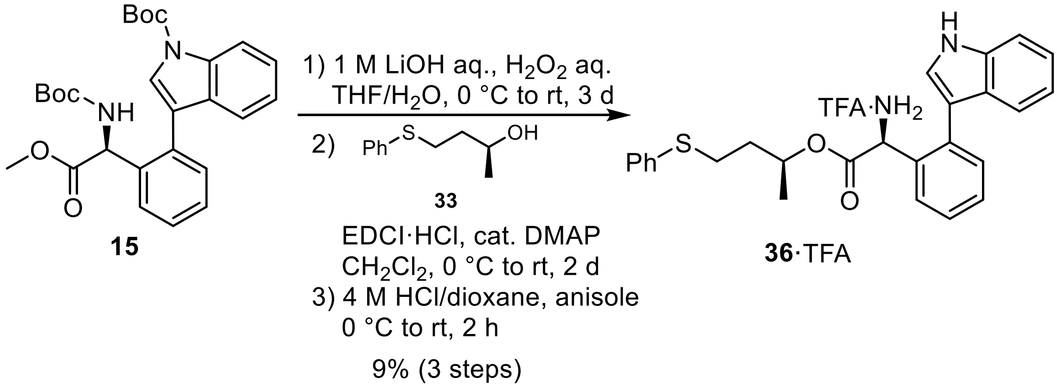

(*S*)-4-(Phenylthio) butan-2-yl (*S*)-2-(2-(1*H*-indol-3-yl)phenyl)-2-aminoacetate (36)

To a solution of **15** (144 mg, 0.300 mmol) in THF (3 mL) was added 30% H_2O_2 aq. (136 L, 1.20 mmol) at room temperature, then added 1.00 M LiOH aq. (600 L, 0.600 mmol) at 0 °C. After stirring at room temperature for 3 days, the reaction mixture was quenched by saturated aqueous NH_4Cl and extracted with EtOAc, then washed with brine. The combined organic layer was dried over $MgSO_4$, then concentrated under reduced pressure to provide the crude carboxylic acid. To the carboxylic acid in CH_2Cl_2 (1.5 mL) were added **33** (54.7 mg, 0.300 mmol) in CH_2Cl_2 (1.5 mL), EDCI·HCl (172 mg, 0.900 mmol) and DMAP (14.7 mg, 0.120 mmol) at 0 °C. After stirring at room temperature for 2 days, the reaction mixture was quenched by saturated aqueous NH_4Cl and extracted with EtOAc, then washed with brine. The combined organic layer was dried over $MgSO_4$, then concentrated under reduced pressure to provide the crude ester. To the crude ester were added anisole (130 L, 1.20 mmol) and 4 M HCl/dioxane (1.50 mL, 6.00 mmol) at 0 °C. The reaction mixture was stirred at room temperature for 2 h, then concentrated under reduced pressure, followed by purification with HPLC to provide the trifluoroacetate salt of the title compound **36** (14.2 mg, 26.1 mol, 9% in 3 steps) as freeze-dried powder: [ ]$_D$ +187.6 (*c* 0.045, $CHCl_3$); t_R = 27.9 min (linear gradient of B in (A + B), 35 to 50% over 45 min); IR(ATR) ν 1681 (CO) cm^{-1}; ^{1}H-NMR (500 MHz, $CDCl_3$) 1.03 (d, *J* = 5.5 Hz, 3H), 1.59–1.69 (m, 2H), 2.28–2.52 (m, 2H), 4.90 (br, 1H), 5.36 (br, 1H), 7.04–7.50 (m, 14H), 8.84 (br, 1H); ^{13}C-NMR (125 MHz, $CDCl_3$) 19.6, 28.9, 35.0, 53.5, 73.3, 111.8, 113.7, 119.3, 120.3, 122.7, 124.5, 126.3, 126.7, 127.4, 128.1, 129.1 (2C), 129.3 (2C), 130.1, 130.6, 132.4, 135.7, 135.9, 136.2, 169.1; HRMS (ESI), *m*/*z* calcd for $C_{26}H_{27}N_2O_2S$ [M+H]$^+$ 431.1788, found 431.1788.

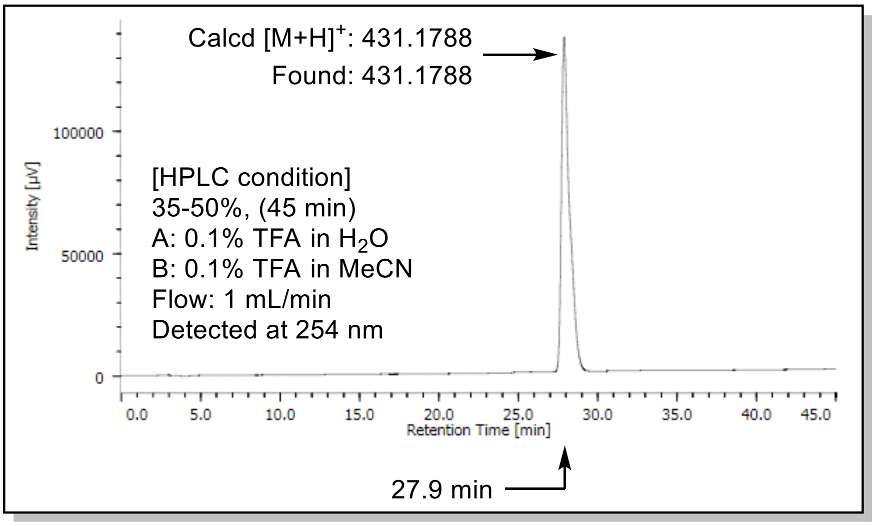

HPLC chart of compound **36**

An MKN-1 derivative, which has a methanesulfonyl group in the place of the methanesulfidyl group (38)

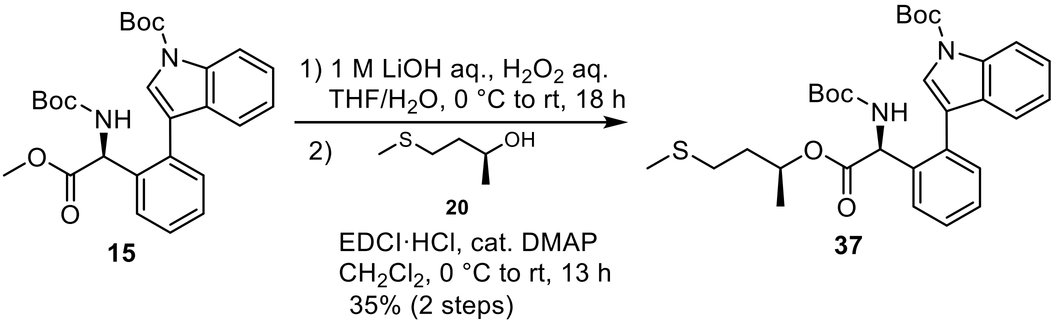

***tert*-Butyl 3-(2-((5*S*,8*S*)-5,12,12-trimethyl-7,10-dioxo-6,11-dioxa-2-thia-9-azatridecan-8-yl) phenyl)-1*H*-indole-1-carboxylate (37)**

To a solution of **15** (144 mg, 0.300 mmol) in THF (3 mL) was added 30% H_2O_2 aq. (136 L, 1.20 mmol) at room temperature, then added 1.00 M LiOH aq. (600 L, 0.600 mmol) at 0 °C. After stirring at room temperature for 18 h, the reaction mixture was quenched by saturated aqueous NH_4Cl and extracted with EtOAc, then washed with brine. The combined organic layer was dried over $MgSO_4$, then concentrated under reduced pressure to provide the crude carboxylic acid. To the carboxylic acid in CH_2Cl_2 (1.5 mL) were added **20** (54.7 mg, 0.300 mmol) in CH_2Cl_2 (1.5 mL), EDCI·HCl (172 mg, 0.900 mmol) and DMAP (14.6 mg, 0.120 mmol) at 0 °C. After stirring at room temperature for 13 h, the reaction mixture was quenched by saturated aqueous NH_4Cl and extracted with EtOAc. The organic layer was dried over $MgSO_4$, then concentrated under reduced pressure, followed by purified by flash column chromatography (EtOAc/hexane = 3/47 to 13/37)

to provide the title compound **37** (60.5 mg, 35% in 2 steps) as white solid: [ ]$_D$ +56.2 (*c* 0.19, $CHCl_3$); IR(ATR) ν 1714 (CO), 1732 (CO) cm^{-1}; ^{1}H-NMR (500 MHz, $CDCl_3$) (mixture of rotamaers) 1.01–1.12 (m, 3H), 1.39 (s, 9H), 1.62 (br, 2H), 1.69 (s, 9H), 1.90–1.96 (m, 3H), 2.00–2.06 (m, 2H), 4.93 (br, 1H), 5.41–5.61 (m, 1H), 7.22–7.45 (m, 7H), 7.89–7.95 (m, 1H), 8.23 (br, 1H); ^{13}C-NMR (125 MHz, $CDCl_3$) (mixture of rotamaers) 15.5, 19.5, 20.0, 28.4 (6C), 29.5, 29.8, 35.3, 35.4, 54.6, 54.7, 71.3, 71.5,79.9, 83.8, 115.4, 119.3, 119.4, 120.0, 120.3, 122.9, 124.7, 125.1, 125.2, 126.8, 128.3,128.4, 130.8, 130.9, 131.7, 133.5, 135.5, 135.6, 136.4, 136.7, 149.9, 154.7, 172.2; HRMS (ESI), *m*/*z* calcd for $C_{31}H_{41}N_2O_6S$ [M+H]$^+$ 569.2680, found 569.2683.

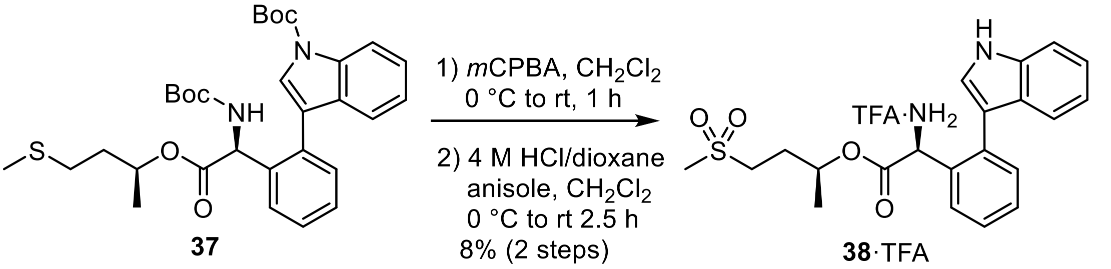

(*S*)-4-(Methylsulfonyl) butan-2-yl (*S*)-2-(2-(1*H*-indol-3-yl)phenyl)-2-aminoacetate (38)

To a solution of **37** (56.9 mg, 0.100 mmol) in CH_2Cl_2 (1 mL) was added *m*CPBA (67.2 mg, 0.300 mmol, ≤77% purity) at 0 °C. After stirring at room temperature for 1 h, the reaction mixture was quenched by saturated aqueous $Na_2S_2O_3$ and extracted with EtOAc, then washed with brine. The combined organic layer was dried over $MgSO_4$, then concentrated under reduced pressure to provide the crude sulfone. To the sulfone were added anisole (44 L, 0.400 mmol) in CH_2Cl_2 (1 mL) and 4 M HCl/dioxane (1.00 mL, 4.00 mmol) at 0 °C. The reaction mixture was stirred at room temperature for 2.5 h, then concentrated under reduced pressure, followed by purification with HPLC to provide the trifluoroacetate salt of the title compound **38** (4.00 mg, 7.77 mol, 8% in 2 steps) as freeze-dried powder: [ ]$_D$ +118.8 (*c* 0.08, MeOH); t_R = 25.4 min (linear gradient of B in (A + B), 20 to 45% over 50 min); IR(ATR) ν 1672 (CO) cm^{-1}; ^{1}H-NMR (500 MHz, MeOH-*d*$_4$) 1.32 (d, 3H), 1.93–2.10 (m, 2H), 2.61–2.75 (m, 2H), 2.84 (s, 3H), 5.14 (br, 1H), 7.12–7.67 (m, 9H), 11.0 (s, 1H); ^{13}C-NMR (125 MHz, MeOH-*d*$_4$) 20.6, 29.9, 41.6, 50.5, 55.2, 74.3, 113.6, 115.4, 120.6, 122.0, 124.2, 126.5, 128.4, 129.2, 130.1, 132.1, 133.4, 134.7, 138.9, 170.5; HRMS (ESI), *m*/*z* calcd for $C_{21}H_{25}N_2O_4S$ [M+H]$^+$ 401.1530, found 401.1528.

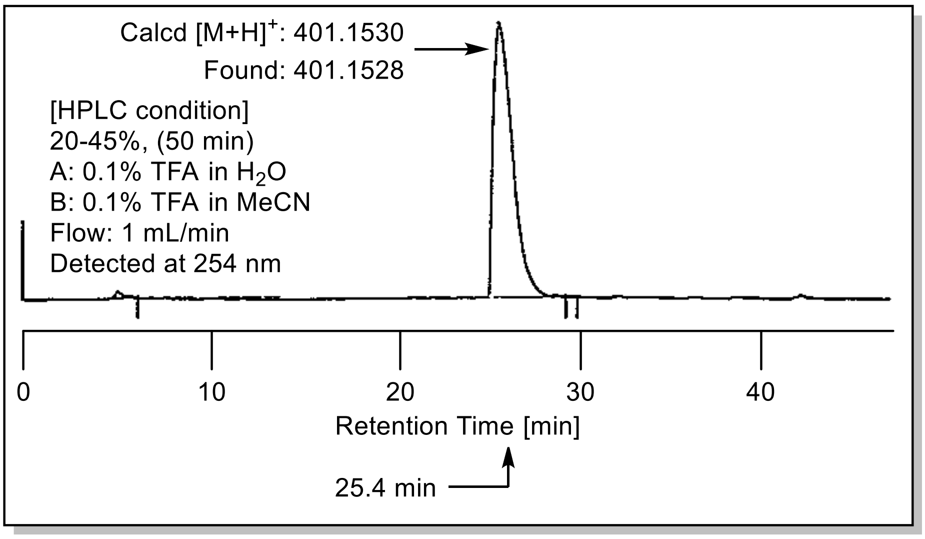

HPLC chart of compound **38**
